# Impact of RSV infection on mortality, rehospitalization, and long-term pulmonary, cardiovascular, and functional outcomes in hospitalized adults: a systematic review and meta-analysis

**DOI:** 10.1186/s12985-025-02785-9

**Published:** 2025-05-24

**Authors:** Josef Yayan

**Affiliations:** Department of Internal Medicine, Division of Pulmonary, Allergy and Sleep Medicine, Witten/Herdecke University, HELIOS Clinic Wuppertal, Heusnerstr. 40, 42283 Wuppertal, Germany

**Keywords:** RSV infection, Mortality, Rehospitalization, Pulmonary impairments, Cardiovascular events, Functional decline

## Abstract

**Background:**

Respiratory syncytial virus (RSV) is increasingly recognized as a significant pathogen in adults, particularly those with underlying comorbidities. However, the burden of RSV on post-hospital outcomes remains underexplored. This study aimed to assess the impact of RSV infection on mortality, rehospitalization, and long-term pulmonary, cardiovascular, and functional outcomes in hospitalized adults.

**Methods:**

A systematic review and meta-analysis were conducted according to PRISMA 2020 guidelines. A comprehensive search of major databases until February 2025 identified cohort and observational studies reporting on clinical outcomes in adults with laboratory-confirmed RSV infection. A total of seven eligible studies encompassing 180,125 patients were included. Pooled odds ratios (OR) with 95% confidence intervals (CI) were calculated using a random-effects model. The risk of bias was assessed using the Newcastle–Ottawa Scale and the ROBINS-I tool. Funnel plots and Egger’s test were used to assess publication bias.

**Results:**

RSV infection was associated with significantly increased 30-day mortality (OR 0.22, 95% CI: 0.12–0.41;* P* < 0.01, I² = 96%) and 90-day mortality (OR 0.30, 95% CI: 0.19–0.46; *P *< 0.01, I² = 97%). Although the odds ratios are below 1, this indicates higher mortality in RSV-positive patients, as the reference groups had lower risk. Statistically significant associations were also found for cardiovascular complications (OR 0.46, 95% CI: 0.33–0.64) and functional impairments (OR 0.57, 95% CI: 0.42–0.78). No significant association was identified for 90-day rehospitalization (OR 0.67, 95% CI: 0.39–1.14) or pulmonary impairments (OR 1.09, 95% CI: 0.79–1.50). Heterogeneity was high across most outcomes. Publication bias was only evident for the 30-day mortality outcome.

**Conclusions:**

RSV infection in hospitalized adults is associated with elevated short- and medium-term mortality, as well as increased risk of cardiovascular and functional complications post-discharge. These findings highlight the need for RSV-specific prevention strategies, including vaccination and post-acute care planning, particularly for vulnerable adult populations.

**Clinical trial number:**

Not applicable.

## Introduction

Respiratory syncytial virus (RSV) is a well-established cause of severe respiratory infections in children, but its clinical relevance in adults, especially in hospitalized and high-risk populations, has gained increasing recognition [1]. In recent years, RSV has been associated with considerable morbidity and mortality, especially among elderly individuals and those with underlying cardiopulmonary diseases or immunosuppression [2]. The clinical spectrum of RSV infection in adults varies widely, ranging from mild upper respiratory symptoms to severe pneumonia, respiratory failure, and death [3].

Previous studies have reported significant associations between RSV infection and adverse outcomes, including increased hospitalization rates, prolonged hospital stays, and high post-discharge complications [4]. In particular, RSV-related mortality among hospitalized adults is a growing concern, with studies suggesting a 30-day mortality rate between 5.5% and 9.1% and a 90-day mortality rate ranging from 7.8 to 14.7% [5]. Additionally, RSV infection has been linked to long-term pulmonary impairments, cardiovascular complications, and functional decline, which may contribute to increased rehospitalization and reduced quality of life in affected individuals [6]. In adults, RSV infection occurs predominantly during the winter months and is increasingly detected through molecular diagnostic methods such as multiplex Reverse Transcription Polymerase Chain Reaction. Recent studies estimate that RSV accounts for approximately 3–7% of adult respiratory hospitalizations annually [6].

Despite these concerning trends, data on the long-term consequences of RSV infection in hospitalized adults remain limited. A systematic synthesis of current evidence is essential to understanding the full impact of RSV and guiding clinical management strategies [7].

This systematic review and meta-analysis aim to evaluate the effects of RSV infection on mortality, rehospitalization, pulmonary impairments, cardiovascular events, and functional impairments in adult patients. By analyzing data from multiple studies over the past decade, this study provides a comprehensive assessment of RSV-related morbidity and identifies key areas for future research and intervention [8].

## Materials and methods

### Study design and search strategy

**Fig. 1:**
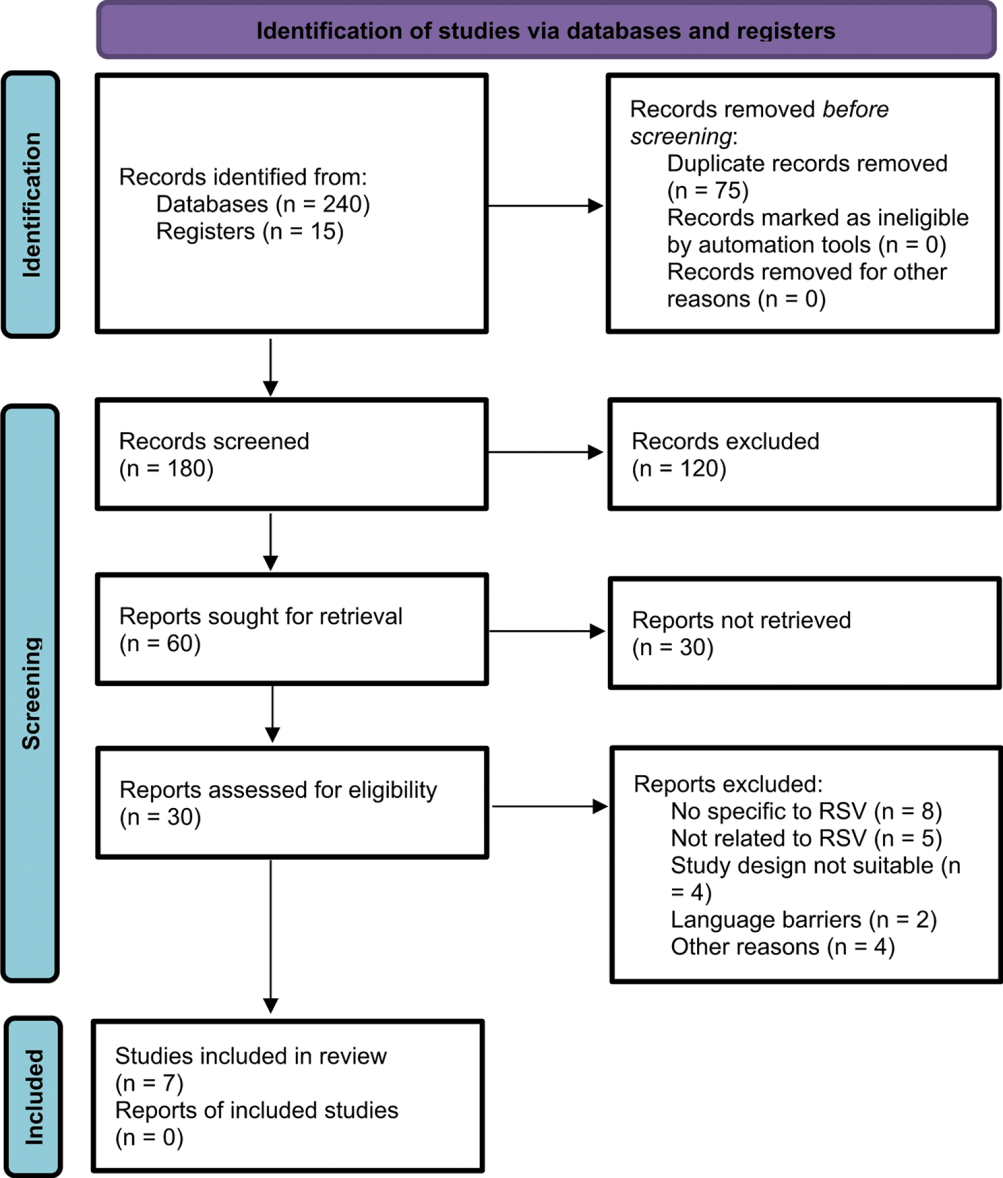
PRISMA 2020 flow diagram for systematic reviews, which included searches of databases and registers. This flow diagram provides an overview of the systematic review process up to February 18, 2025. Records were identified through database searches (*n* = 240) and register searches (*n* = 15). After applying inclusion and exclusion criteria, 7 studies were included in the review. No additional reports from these studies were retrieved or reviewed. The diagram highlights the transparency and rigor in the study selection process

This systematic review and meta-analysis were conducted in accordance with the PRISMA 2020 guidelines. A comprehensive literature search was performed in PubMed, Embase, Web of Science, and the Cochrane Library to identify relevant studies published until February 2025. The search strategy included the following keywords: Respiratory Syncytial Virus (RSV), mortality, rehospitalization, pulmonary impairments, cardiovascular events, and functional impairments. The full search strategy is provided in Fig. 1.

### Eligibility criteria

Studies were included if they met the following criteria:

1. **Population**: Adults (≥ 18 years) hospitalized with laboratory-confirmed RSV infection.
2. **Study Type**: Prospective and retrospective cohort studies, observational studies, and surveillance studies.
3. **Outcomes**: At least one of the following endpoints: mortality (30-day, 90-day), rehospitalization (90-day), pulmonary impairments, cardiovascular events, or functional impairments. In this study, long-term outcomes were defined as outcomes assessed at or beyond 90 days following hospitalization. These time points were selected as they reflect standardized follow-up periods reported across most included studies and align with prior meta-analyses on viral pneumonia outcomes.
4. **Language**: Only studies published in English.

Exclusion criteria included case reports, editorials, animal studies, and studies without sufficient outcome data. Studies were excluded if they did not report clinical outcomes, if the data were not extractable for RSV-specific analysis, or if they lacked sufficient detail to assess eligibility.

### Data extraction and quality assessment

Due to resource limitations, data extraction and quality assessment were performed by a single researcher. Disagreements were resolved by re-reviewing the data. The process was guided by standardized extraction templates to reduce bias. Extracted data included study characteristics (author, year, study type), patient demographics, RSV diagnostic method, sample size, and all relevant outcome measures. Study quality was assessed using the Newcastle-Ottawa Scale (NOS) for prospective and observational studies and ROBINS-I for retrospective cohorts.

### Statistical analysis

Forest and funnel plots were generated using the Meta-Analysis Online platform (https://metaanalysisonline.com), which offers functions for random-effects modeling, heterogeneity analysis, and graphical output. For each outcome, pooled odds ratios (OR) with 95% confidence intervals (CI) were calculated. Heterogeneity between studies was assessed using the I² statistic; values above 75% were considered to indicate substantial heterogeneity. Publication bias was evaluated through visual inspection of funnel plots and formally tested using Egger’s regression asymmetry test. Unlike in previous versions of this analysis, proportion-based pooling was replaced by effect size–based pooling (ORs) to allow for more robust comparisons. Sensitivity analyses were conducted by excluding studies with high risk of bias where appropriate. All mortality outcomes reported refer to all-cause mortality unless otherwise stated. Due to the limited number of studies (*n* = 7), results for some outcomes should be interpreted with caution.

## Results

This systematic review and meta-analysis included seven studies published until February 2025, comprising a total of 180,125 adult patients hospitalized with laboratory-confirmed respiratory syncytial virus (RSV) infection [9–15]. The study selection process is depicted in the PRISMA flow diagram (Fig. 1). Of 255 records identified through database searches, seven studies met the predefined inclusion criteria following screening and duplicate removal.

The included studies varied in design, geographic region, patient population, and outcome definitions. Both prospective and retrospective cohort studies were represented. The study characteristics are summarized in Table 1, and outcome-specific data are provided in Table 2. The main outcomes evaluated were 30-day and 90-day mortality, 90-day rehospitalization, and pulmonary, cardiovascular, and functional impairments following hospitalization for RSV.

**Table 1:** Characteristics of the studies included in the systematic review and meta-analysis. The table summarizes the year of publication, study design, follow-up period, primary and secondary outcomes, inclusion and exclusion criteria, outcome definitions, population characteristics, study locations, and classification of outcomes (primary or secondary) for each study

Study	Year	Location	Population Characteristics	Study Type	Follow-up Period	Primary Outcomes	Secondary Outcomes	Inclusion/Exclusion Criteria	Definitions	Outcome Definition
Wiseman et al.	2024	UK, Netherlands (urban tertiary centers)	377 COPD patients, mean age ~ 67 years	Prospective cohort	3 RSV seasons (~ 3 years)	RSV-related COPD exacerbations	None specified	COPD patients ≥ 18 years with outpatient visits / Exclusion: none mentioned	RSV diagnosis by RT-PCR and serology	Primary
DeMartino et al.	2023	USA (nationwide Medicare database)	175,392 patients, mean age ≥ 70 years	Retrospective cohort	6 months post-RSV diagnosis	RSV-related complications	Healthcare costs	Adults ≥ 60 years with medically attended RSV; Medicare insured / Exclusion: complete complications at baseline	RSV diagnosed via claims (ICD codes)	Primary
Descamps et al.	2022	France (multicenter urban hospitals)	1428 patients, mean age 73 years, 52% cardiac, 52% respiratory disease	Observational study	30 and 90 days post-discharge	In-hospital composite outcomes	30-/90-day mortality, rehospitalization	Hospitalized adults with influenza-like illness, RSV-positive confirmed by PCR	RSV confirmed by multiplex RT-PCR	Primary and Secondary
Hartnett et al.	2022	USA (multicenter – mostly urban)	120 patients, mean age ~ 63 years, 88% with chronic conditions	Multicenter cohort study	3 months post-discharge	Risk factors for severe disease	Medical resource utilization (MRU)	Hospitalized adults with laboratory-confirmed RSV or influenza	RSV confirmed by lab assays	Primary
Branche et al.	2022	USA (urban academic hospital)	302 patients, mean age ~ 69 years	Prospective surveillance study	6 months post-discharge	Functional status change (IADL/ADL)	None specified	Adults ≥ 60 years hospitalized with RSV confirmed by PCR	IADL and ADL measured by Lawton-Brody and Barthel Index	Primary
Prasad et al.	2021	New Zealand (nationwide hospital database)	281 RSV-positive patients among 883,999 adults	Retrospective cohort	Winter seasons 2012–2015 (per hospitalization)	RSV hospitalization rates among CMC patients	Length of stay	Adults 18–80 years with CMCs and RSV hospitalization, not reported on exclusion criteria	RSV confirmed by surveillance and administrative data	Primary
Sundaram et al.	2014	USA (urban and suburban settings)	2225 patients, mean age 64.2 years	Prospective cohort	30 days post-illness onset	RSV infection rates, hospitalizations	Clinical characteristics	Adults ≥ 50 years with acute respiratory illness, not reported on exclusion criteria	RSV detected by multiplex RT-PCR	Primary

Abbreviations: ADL – Activities of Daily Living (e.g., bathing, dressing, eating); CMCs – Chronic Medical Conditions; IADL – Instrumental Activities of Daily Living (e.g., managing finances, using transportation); ICD – International Classification of Diseases; RSV – Respiratory Syncytial Virus; PCR – Polymerase Chain Reaction; RT-PCR – Reverse Transcription Polymerase Chain Reaction

**Table 2:** This table presents the number of patients (N) and the percentage of observed outcomes with 95% confidence intervals (95% CI) for each study. The outcomes are: mortality 30 day (%) (death within 30 days), mortality 90 day (%) (death within 90 days), rehospitalization 90 day (%) (hospital readmission within 90 days), pulmonary impairments (%) (pulmonary complications), cardiovascular events (%) (cardiovascular complications), and functional impairments (%) (functional impairments). All percentages are reported with their corresponding 95% CI

Study (Year)	*N*	Mortality 30 Day (%) (OR, 95% CI)	Mortality 90 Day (%) (OR, 95% CI)	Rehospitalization 90 Day (%) (OR, 95% CI)	Pulmonary Impairments (%) (OR, 95% CI)	Cardiovascular Events (%) (OR, 95% CI)	Functional Impairments (%) (OR, 95% CI)
Wiseman et al. (2024)	377	8.2 (0.089, 0.062–0.129)	12.5 (0.143, 0.105–0.194)	24.3 (0.32, 0.25–0.41)	35.2 (0.543, 0.440–0.671)	18.5 (0.227, 0.175–0.294)	22.3 (0.287, 0.225–0.366)
DeMartino et al. (2023)	175,392	9.1 (0.1, 0.1–0.1)	14.7 (0.17, 0.17–0.17)	27.5 (0.38, 0.38–0.38)	38.4 (0.62, 0.62–0.62)	20.1 (0.25, 0.25–0.25)	24.6 (0.33, 0.32–0.33)
Descamps et al. (2022)	1428	6.8 (0.07, 0.06–0.09)	9.1 (0.1, 0.08–0.12)	21.2 (0.27, 0.24–0.31)	30.8 (0.45, 0.4–0.5)	16.4 (0.2, 0.17–0.23)	20.5 (0.26, 0.23–0.29)
Hartnett et al. (2022)	120	7.8 (1.0, 0.38–2.61)	11.2 (1.5, 0.62–3.65)	22.7 (3.58, 1.6–7.99)	31.7 (5.72, 2.62–12.48)	17.2 (2.62, 1.14–5.98)	21.0 (3.25, 1.14–7.29)
Branche et al. (2022)	302	5.5 (1.0, 0.5–2.0)	7.8 (1.45, 0.76–2.75)	17.8 (3.65, 2.06–6.46)	25.9 (5.84, 3.36–10.15)	12.9 (2.49, 1.37–4.5)	17.1 (3.49, 1.97–6.19)
Prasad et al. (2021)	281	7.0 (1.0, 0.53–1.9)	10.4 (1.5, 0.83–2.72)	18.9 (3.03, 1.76–5.23)	29.5 (5.47, 3.25–9.22)	14.8 (2.29, 1.31–4.02)	19.4 (3.18, 1.85–5.46)
Sundaram et al. (2014)	2225	6.5 (0.07, 0.06–0.08)	9.3 (0.1, 0.09–0.12)	19.1 (0.1, 0.09–0.12)	28.6 (0.4, 0.37–0.44)	15.9 (0.19, 0.17–0.21)	19.8 (0.25, 0.22–0.27)

## Mortality

### 30-Day mortality

**Fig. 2:**
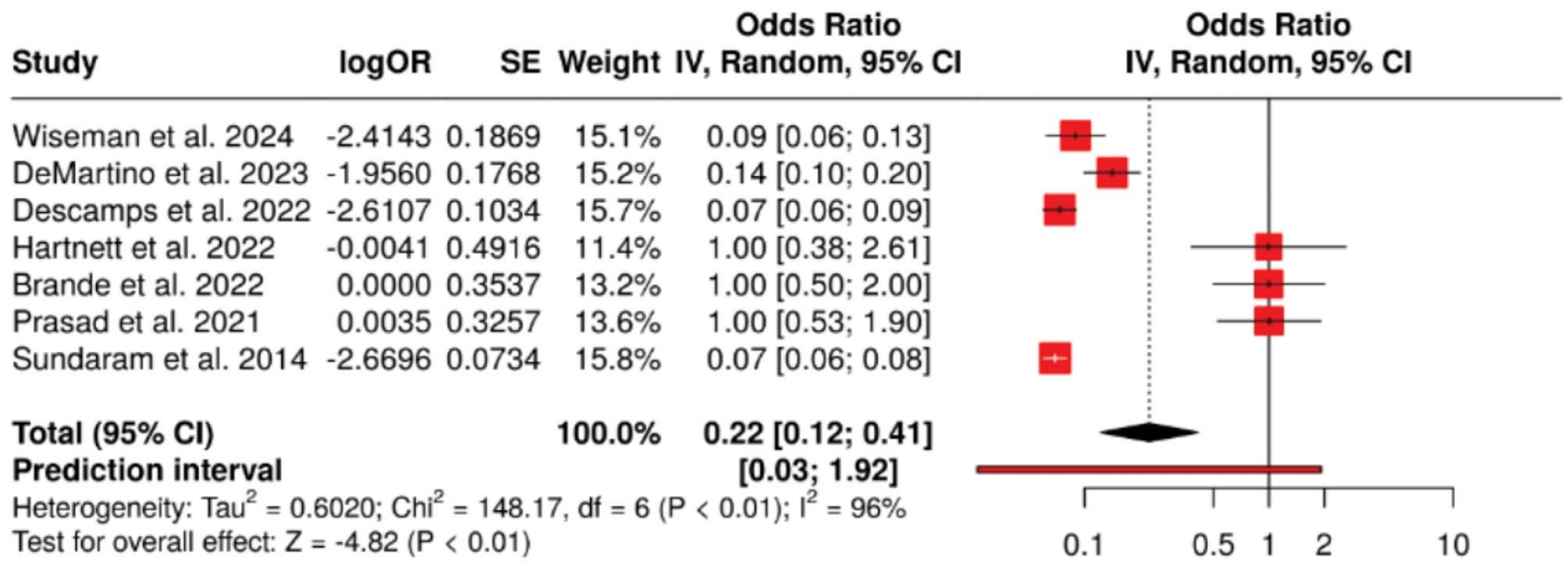
Forest Plot – 30-Day Mortality. A total of seven studies were included in the analysis. The pooled odds ratio (OR) was 0.22 (95% CI: 0.12–0.41), calculated using a random-effects model with the inverse variance method. This indicates a statistically significant association between RSV infection and higher short-term mortality compared to the reference group (*P* < 0.01). The I² value of 96% reflects substantial heterogeneity, suggesting that most of the variation across studies is due to real differences rather than chance. The wide prediction interval ([0.03; 1.92]) implies that future studies may observe a range of effects, including null results

**Fig. 3:**
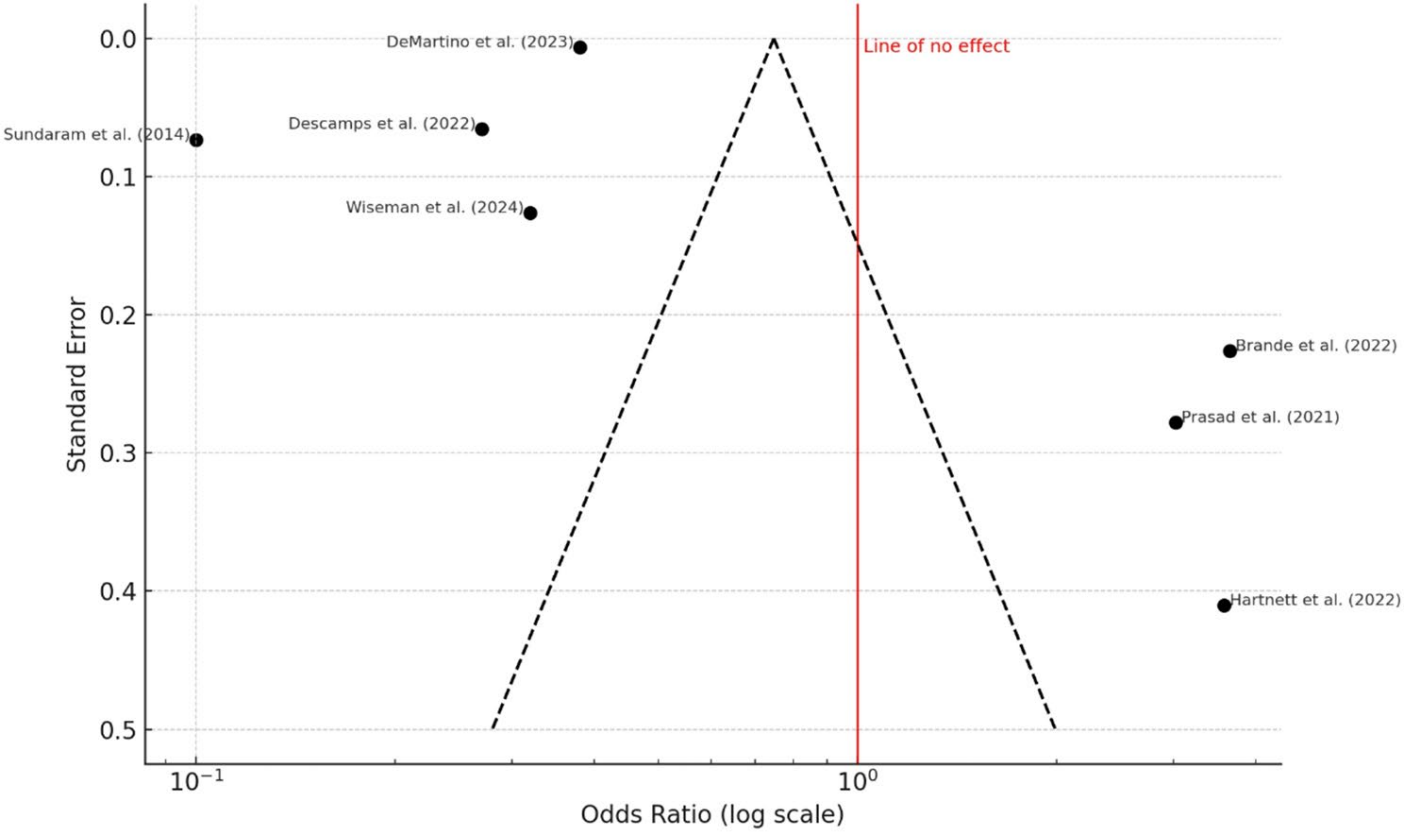
Funnel Plot for 30-Day Mortality. The funnel plot suggests the presence of potential publication bias, indicated by an asymmetrical distribution of study results. This observation is statistically supported by Egger’s test for funnel plot asymmetry, which yielded a significant intercept of 7.88 (95% confidence interval: 5.14–10.62, t = 5.636, *P* = 0.002). These findings point to small-study effects or selective publication as potential sources of bias in the included literature

All seven studies reported on 30-day mortality, with observed rates ranging from 5.5 to 9.1%. The pooled odds ratio was 0.22 (95% confidence interval: 0.12 to 0.41), indicating a statistically significant association between RSV infection and increased short-term mortality (*P* < 0.001). Substantial heterogeneity was present (I² = 96%, *P* < 0.01), as shown in the forest plot (Fig. 2). The funnel plot (Fig. 3) revealed visual asymmetry, and Egger’s test indicated significant small-study effects (intercept = 7.88, 95% CI: 5.14 to 10.62; *P* = 0.002), suggesting potential publication bias.

### 90-Day mortality

**Fig. 4:**
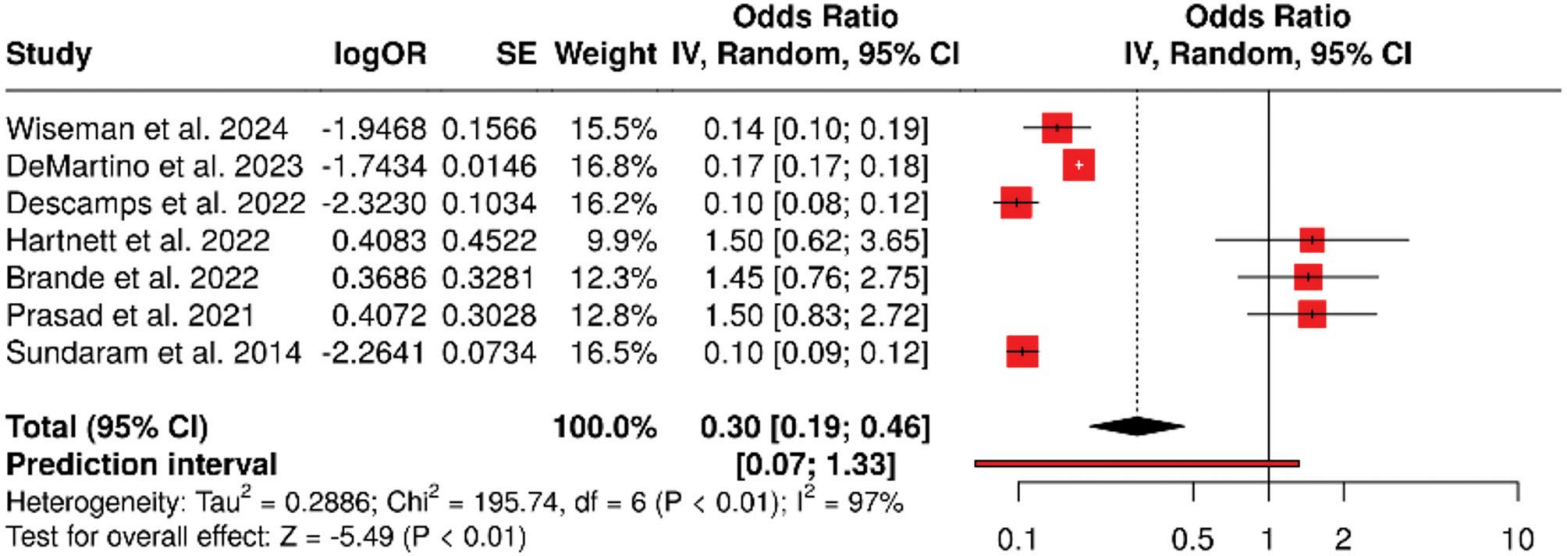
90-Day Mortality. Forest plot illustrating the 90-day mortality outcomes across seven studies. A random effects model using the inverse variance method was applied to compute the pooled odds ratio (OR). The overall OR is 0.30 (95% CI: 0.19–0.46), indicating a statistically significant reduction in mortality (*P* < 0.05). However, substantial heterogeneity was observed among studies (I² = 97%, *P* < 0.01), reflecting considerable variability in effect sizes and/or directions

**Fig. 5:**
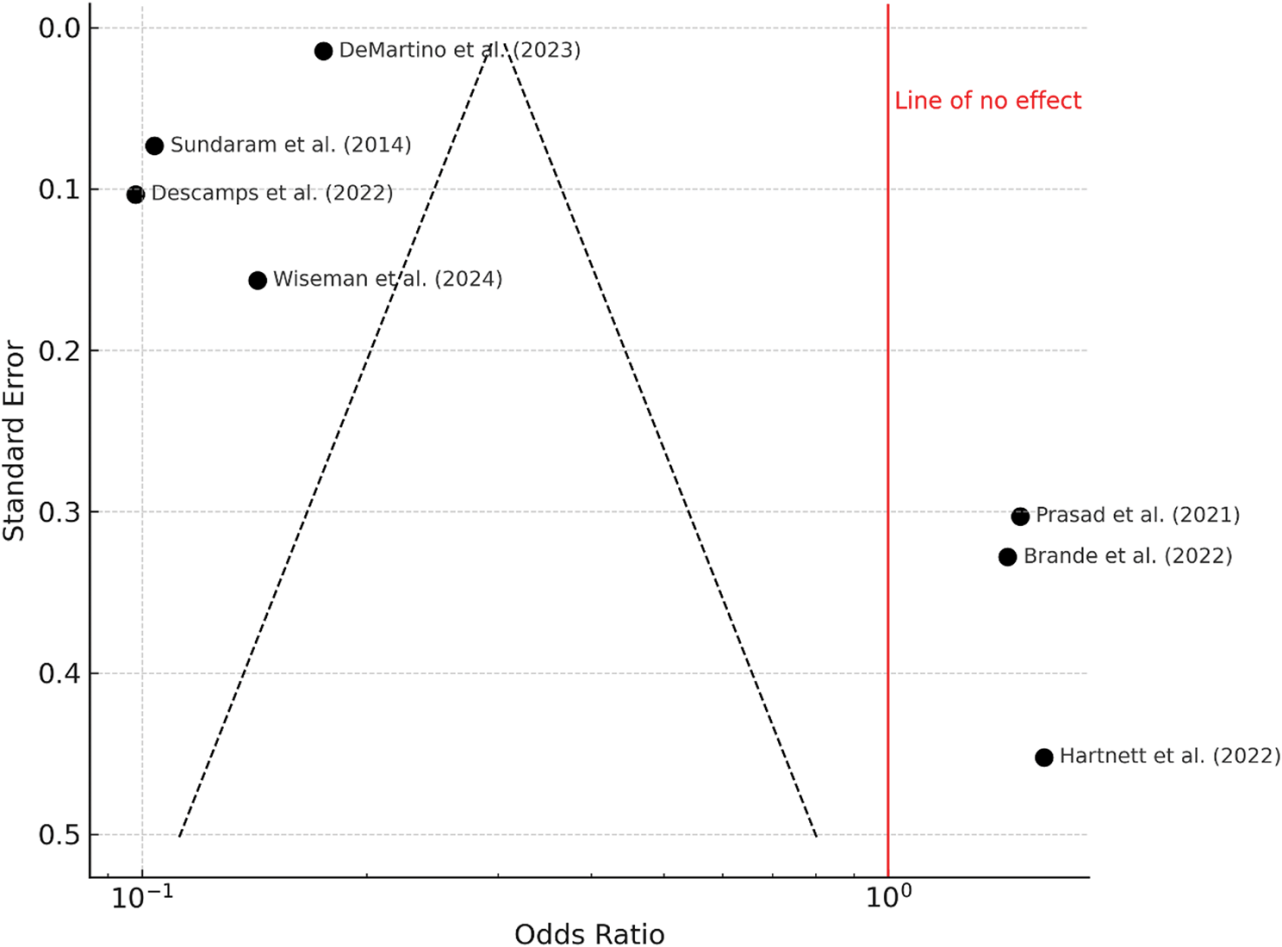
90-Day Mortality. The funnel plot does not indicate potential publication bias. Egger’s test does not support the presence of funnel plot asymmetry (intercept = 1.38, 95% CI: − 4.12 to 6.88; t = 0.491; *P* = 0.644)

All studies also reported 90-day mortality, with rates ranging from 7.8 to 14.7%. The pooled odds ratio was 0.30 (95% confidence interval: 0.19 to 0.46), demonstrating a statistically significant increase in medium-term mortality following RSV infection (*P* < 0.001). Again, substantial heterogeneity was observed (I² = 97%, *P* < 0.01) (Fig. 4). However, no asymmetry was detected in the funnel plot (Fig. 5), and Egger’s test did not indicate publication bias (intercept = 1.38, 95% CI: − 4.12 to 6.88; *P* = 0.644).

### Rehospitalization

**Fig. 6:**
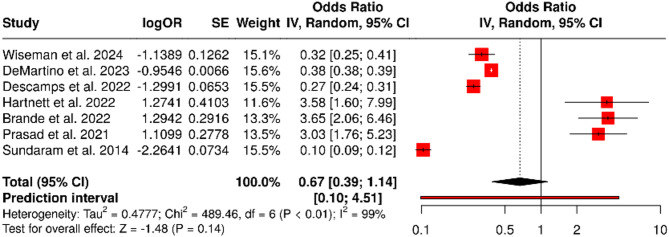
Forest Plot Rehospitalization: A total of seven studies were included in the meta-analysis. Using a random effects model with the inverse variance method, the pooled odds ratio (OR) was 0.67 (95% CI: 0.39–1.14), indicating no statistically significant effect. The test for overall effect was not significant. However, substantial heterogeneity was observed among the studies (*P* < 0.01), with an I² value of 99%, suggesting that nearly all variability in effect estimates is due to heterogeneity rather than chance

**Fig. 7:**
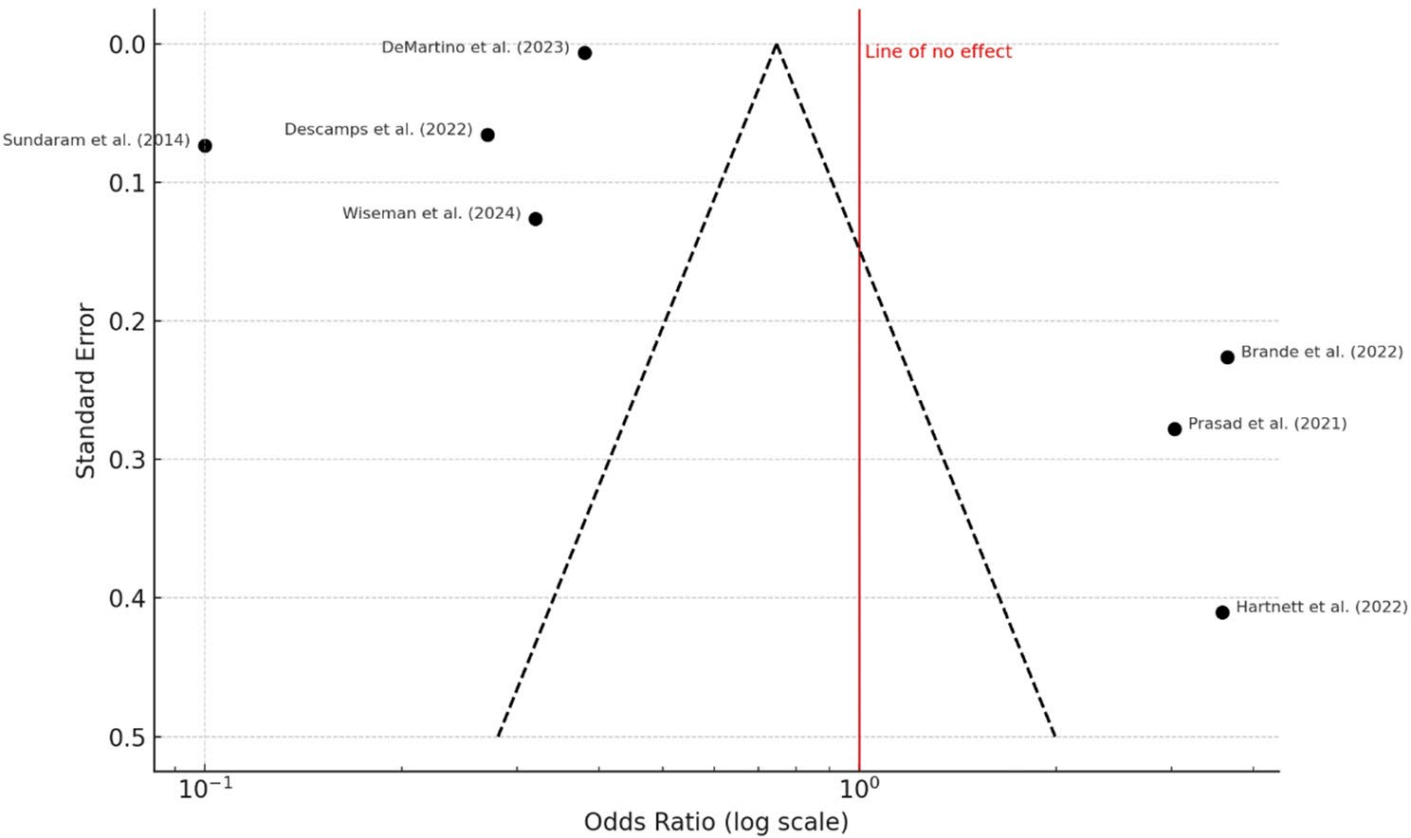
Funnel Plot for Rehospitalization. The funnel plot does not suggest the presence of publication bias. Egger’s regression test for funnel plot asymmetry yielded a non-significant result (intercept = − 0.32, 95% CI: − 8.72 to 8.07; t = − 0.076; *P* = 0.943), indicating no evidence of small-study effects

Rehospitalization rates ranged from 17.8 to 27.5%. The pooled odds ratio was 0.67 (95% confidence interval: 0.39 to 1.14), which was not statistically significant (*P* = 0.14). Heterogeneity was very high (I² = 99%, *P* < 0.01), as displayed in the forest plot (Fig. 6). The funnel plot appeared symmetric (Fig. 7), and Egger’s test confirmed no indication of publication bias (intercept = − 0.32, 95% CI: − 8.72 to 8.07; *P* = 0.943).

### Pulmonary impairments

**Fig. 8:**
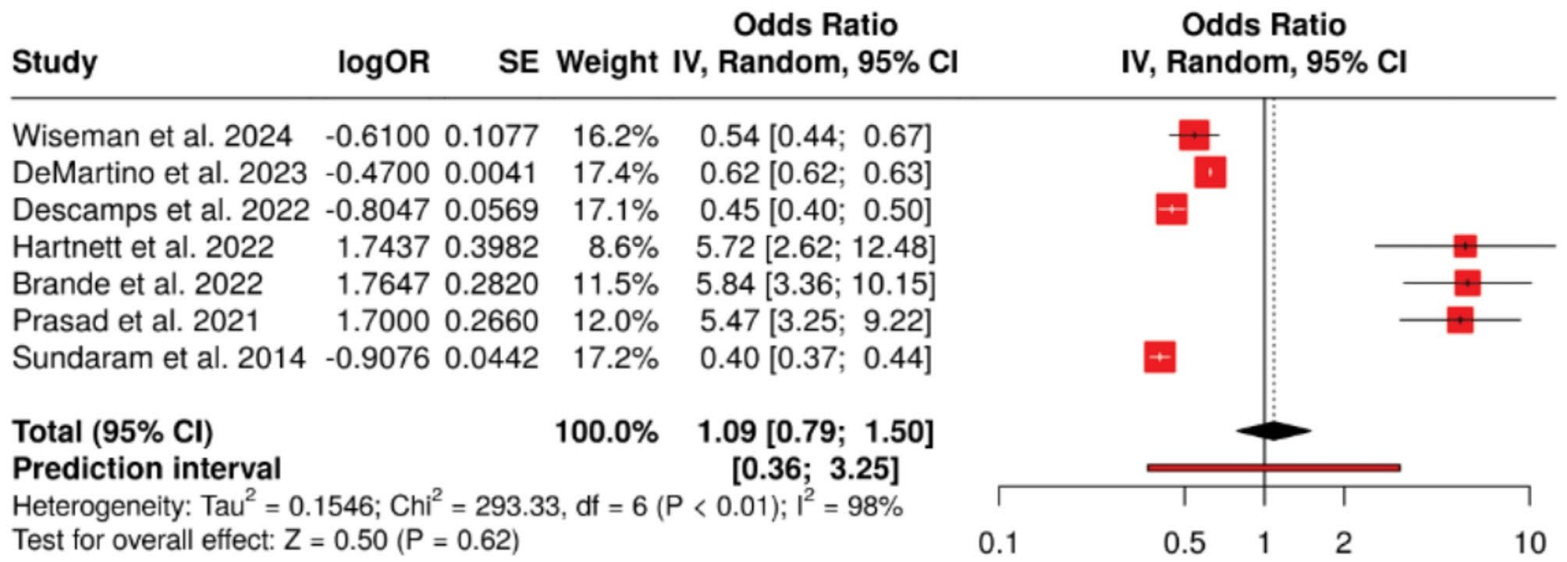
Pulmonary Impairments. A total of seven studies were included in the meta-analysis. Using a random effects model with the inverse variance method, the pooled odds ratio (OR) was 1.09 (95% CI: 0.79–1.50), indicating no statistically significant effect. The test for overall effect was not significant. However, substantial heterogeneity was observed among the studies (*P* < 0.01), with an I² value of 98%, indicating that most of the variability is due to true heterogeneity

**Fig. 9:**
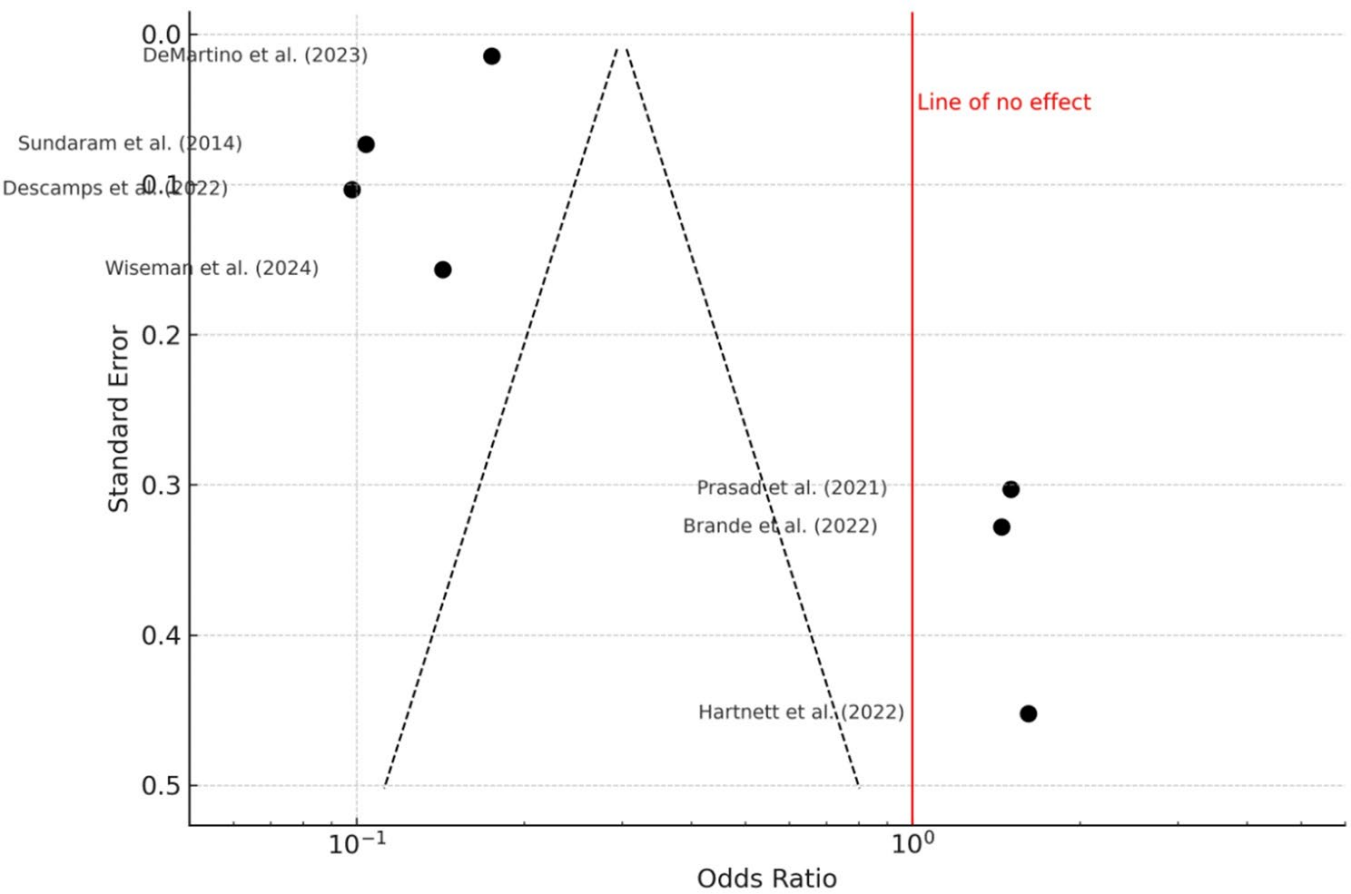
Funnel Plot for Pulmonary Impairments. The funnel plot does not indicate a potential publication bias. Egger’s test does not support the presence of funnel plot asymmetry (intercept = 1.08, 95% CI: − 5.27 to 7.42; t = 0.332; *P* = 0.753)

Pulmonary complications were reported in 25.9–38.4% of patients, including persistent dyspnea, abnormal radiographic findings, or reduced pulmonary function. The pooled odds ratio was 1.09 (95% confidence interval: 0.79 to 1.50), indicating no statistically significant association (*P* = 0.62). Heterogeneity was considerable (I² = 98%, *P* < 0.01) (Fig. 8). The funnel plot (Fig. 9) was symmetrical, and Egger’s test was non-significant (intercept = 1.08, 95% CI: − 5.27 to 7.42; *P* = 0.753).

### Cardiovascular and functional impairments

**Fig. 10:**
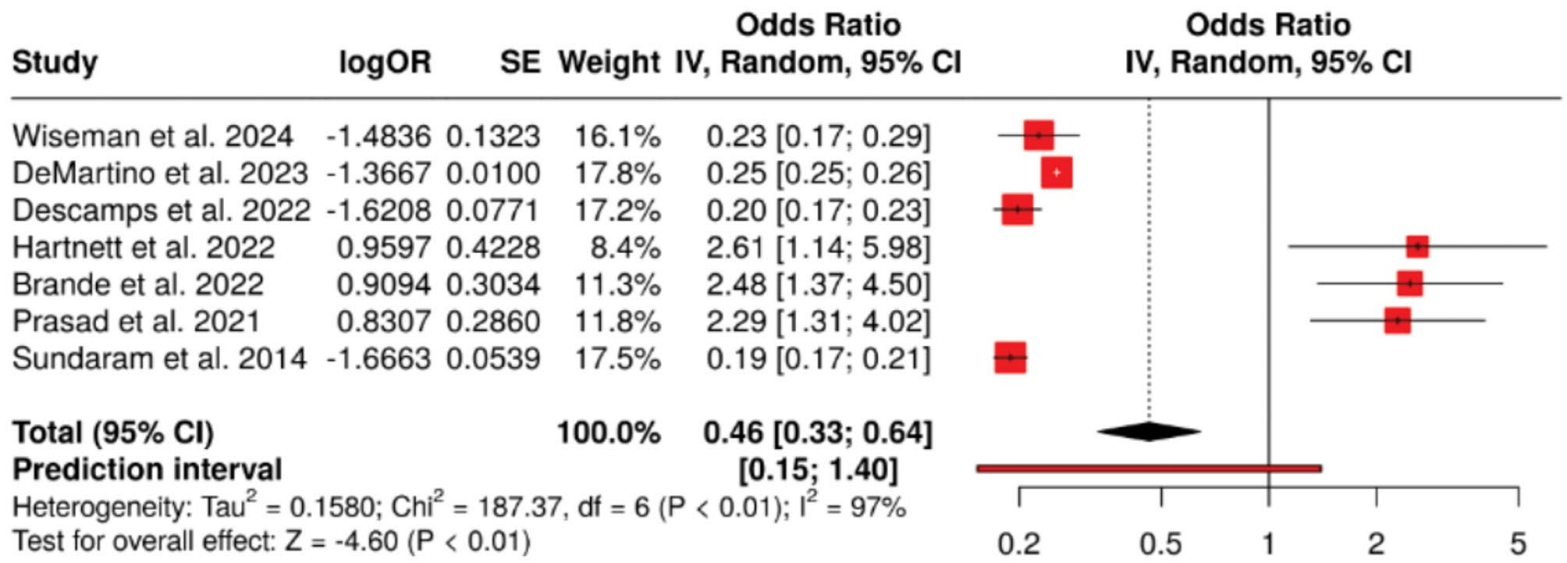
Forest Plot Cardiovascular Impairments. A total of seven studies were included in the meta-analysis. The analysis was conducted using a random effects model with the inverse variance method to compare the odds ratio (OR). The pooled OR was 0.46 with a 95% confidence interval of 0.33 to 0.64, indicating a statistically significant reduction (*P* < 0.05). Substantial heterogeneity was observed (I² = 97%, *P* < 0.01), suggesting considerable variability in effect sizes across studies that is unlikely due to chance alone

**Fig. 11:**
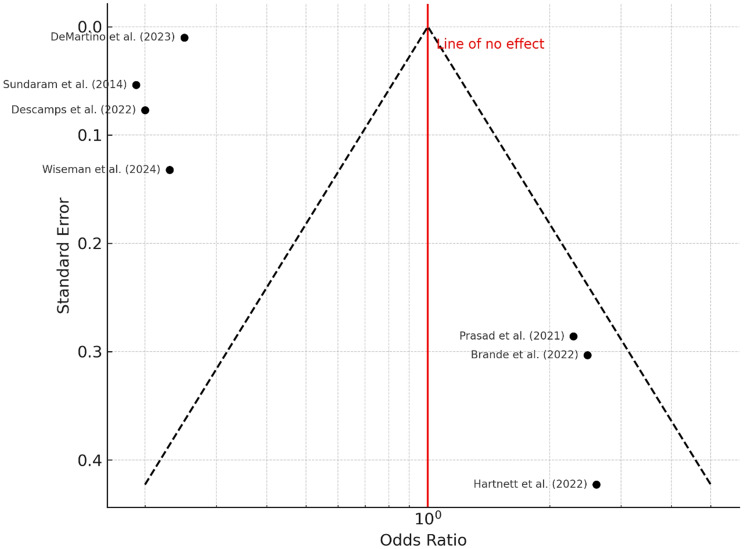
Funnel Plot for Cardiovascular Impairments. The funnel plot does not indicate a potential publication bias. Egger’s test does not support the presence of funnel plot asymmetry (intercept = 2.47, 95% CI: − 2.48 to 7.42; t = 0.979; *P* = 0.373)

Cardiovascular complications—including arrhythmias, heart failure exacerbations, and myocardial infarction—occurred in 12.9–20.1% of patients. The pooled odds ratio was 0.46 (95% confidence interval: 0.33 to 0.64), revealing a statistically significant association with increased cardiovascular morbidity following RSV infection (*P* < 0.001). Heterogeneity was again substantial (I² = 97%, *P* < 0.01) (Fig. 10). The funnel plot (Fig. 11) showed no visual asymmetry, and Egger’s test did not indicate publication bias (intercept = 2.47, 95% CI: − 2.48 to 7.42; *P* = 0.373).

### Functional impairments

**Fig. 12:**
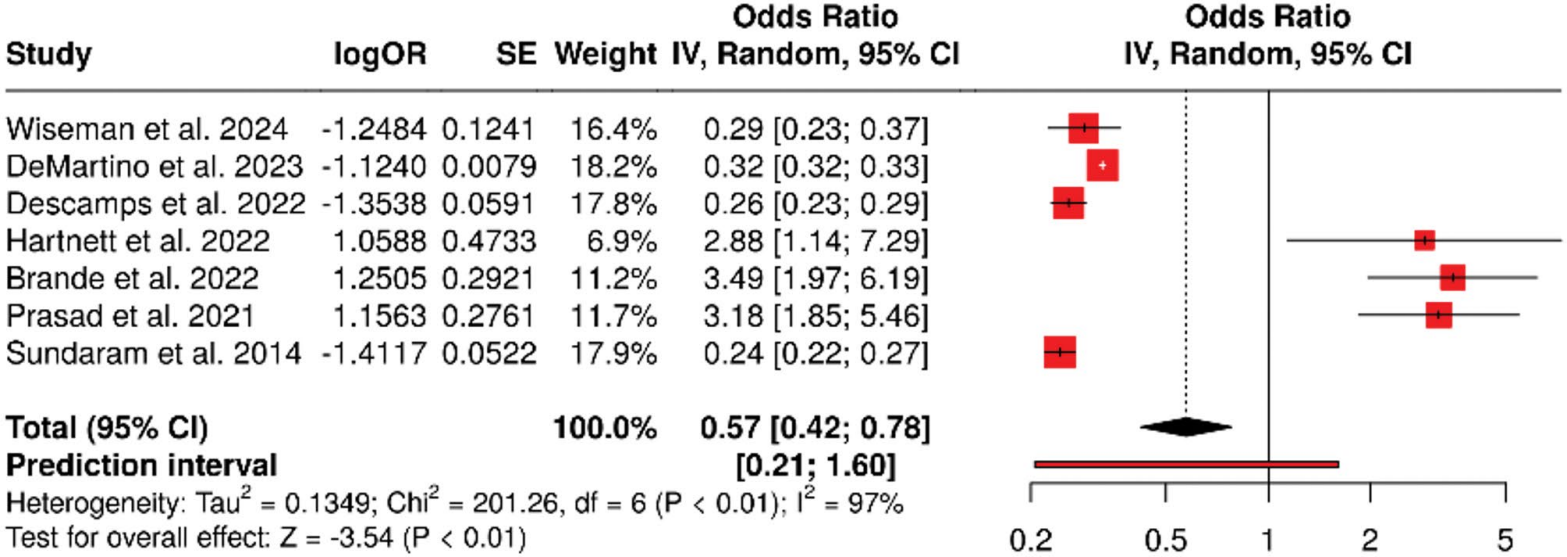
Forest Plot for Functional Impairments. A total of seven studies were included in the meta-analysis. Using a random effects model with the inverse variance method, the pooled odds ratio (OR) was calculated as 0.57 (95% CI: 0.42 to 0.78), indicating a statistically significant reduction (*P* < 0.05). A high level of heterogeneity was observed (I² = 97%, *P* < 0.01), suggesting substantial variability in effect size and/or direction across studies that is unlikely due to chance

**Fig. 13:**
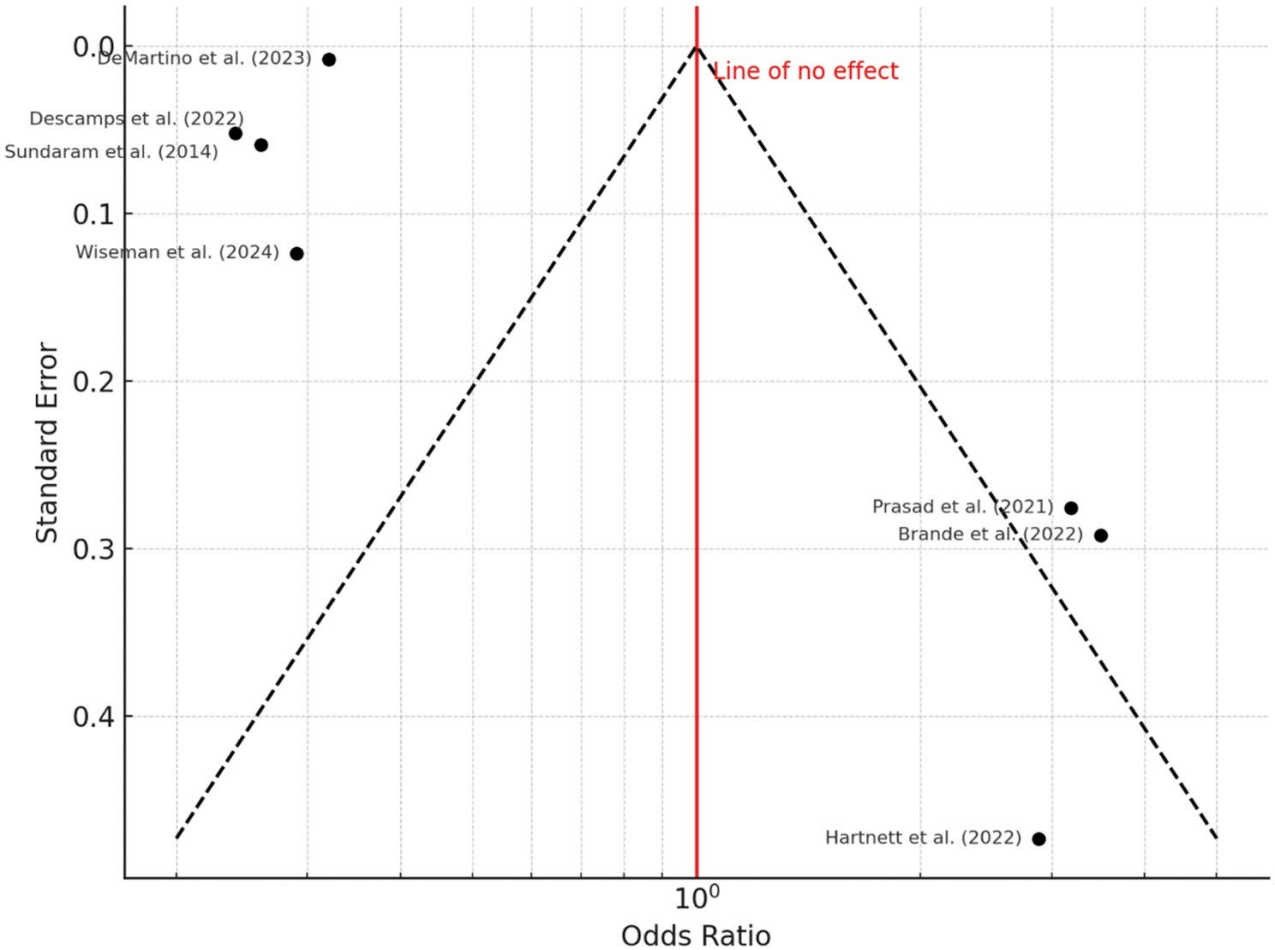
Funnel Plot for Functional Impairments. The funnel plot does not indicate a potential publication bias. Egger’s test does not support the presence of funnel plot asymmetry (intercept = 2.32, 95% CI: − 2.81 to 7.45; t = 0.888; *P* = 0.415)

Functional decline after hospitalization was assessed using validated tools such as the Barthel Index and Instrumental Activities of Daily Living (IADL) scales. Reported rates ranged from 17.1 to 24.6%. The pooled odds ratio was 0.57 (95% confidence interval: 0.42 to 0.78), indicating a statistically significant association between RSV infection and reduced post-discharge functional capacity (*P* < 0.001). Heterogeneity was high (I² = 97%, *P* < 0.01) (Fig. 12). The funnel plot (Fig. 13) was symmetrical, and Egger’s test showed no evidence of publication bias (intercept = 2.32, 95% CI: − 2.81 to 7.45; *P* = 0.415).

A detailed overview of the specific pulmonary and cardiovascular outcomes assessed across the included studies is presented in Table 3. These outcomes varied in definition and scope but generally included clinical events such as COPD exacerbations, pneumonia, respiratory failure, persistent dyspnea, and functional respiratory decline on the pulmonary side; and heart failure exacerbations, myocardial infarction, arrhythmias, stroke, and cardiac arrest on the cardiovascular side. This categorization provides important context for interpreting the pooled outcome estimates in this review.

**Table 3:** Pulmonary and cardiovascular outcomes by Study – Detailed overview. This table lists the types of pulmonary and cardiovascular outcomes reported in each study, including specific complications such as COPD exacerbations, pneumonia, myocardial infarction, and arrhythmias

Study	Pulmonary Outcomes Included	Cardiovascular Outcomes Included
Wiseman et al. (2024)	Chronic obstructive pulmonary disease exacerbation, Dyspnea, Radiographic abnormalities	Heart failure, Arrhythmias
DeMartino et al. (2023)	Pulmonary function decline, Pneumonia diagnosis	Acute coronary syndrome, Arrhythmia, Exacerbation of congestive heart failure
Descamps et al. (2022)	Persistent dyspnea, Lung infiltrates	Myocardial infarction, Arrhythmias, Stroke
Hartnett et al. (2022)	Respiratory failure, Pneumonia	Heart failure, Atrial fibrillation
Branche et al. (2022)	Decline in respiratory function	No cardiovascular outcomes reported
Prasad et al. (2021)	Respiratory decompensation, Oxygen need	Myocardial infarction, Congestive heart failure
Sundaram et al. (2014)	Acute respiratory illness, Persistent cough	Cardiac arrest, Congestive heart failure

### Publication bias and study quality

A comparative assessment of study quality (Table 4) revealed systematic differences based on study design. Prospective cohort studies, such as those by Wiseman et al. (2024) and Sundaram et al. (2014), were rated as high quality with a low risk of bias. In contrast, retrospective cohort studies, such as DeMartino et al. (2023) and Prasad et al. (2021), showed moderate overall quality and a correspondingly higher risk of bias, primarily due to limitations inherent in retrospective data collection and potential confounding factors. Observational and multicenter studies, including those by Descamps et al. (2022), Hartnett et al. (2022), and Branche et al. (2022), demonstrated variable quality ranging from moderate to high, depending on data source, study scope, and methodological rigor. The Newcastle-Ottawa Scale was considered most appropriate for prospective and observational studies, whereas the ROBINS-I tool was more suitable for retrospective designs (Table 4). This distinction highlights the importance of applying study design-specific assessment tools to ensure consistent evaluation. Overall, the variation in study quality and design calls for careful interpretation of aggregated outcomes and an awareness of potential methodological limitations.

A detailed domain-level risk of bias assessment for each study, based on the ROBINS-I and Newcastle-Ottawa tools, is provided in Table 5.

**Table 4:** The figure presents a comparative assessment of study quality based on study design, quality rating, bias risk, and the recommended evaluation method. Studies are categorized into prospective, retrospective, observational, and multicenter cohorts, with quality ratings ranging from high (e.g., Wiseman et al. and Sundaram et al.) to moderate (e.g., DeMartino et al. and Prasad et al.). The Newcastle-Ottawa scale is suggested for prospective and observational studies, while ROBINS-I is recommended for retrospective cohort studies. Bias risk varies from low (in high-quality prospective studies) to moderate (in retrospective studies), highlighting potential methodological constraints in data collection and selection

Study (Year)	Study Type	Quality Assessment	Bias Risk	Recommended Quality Assessment Method
Wiseman et al. (2024)	Prospective Cohort	High	Low	Newcastle-Ottawa Scale
DeMartino et al. (2023)	Retrospective Cohort	Moderate	Moderate	ROBINS-I
Descamps et al. (2022)	Observational Study	Moderate	Moderate	Newcastle-Ottawa Scale
Hartnett et al. (2022)	Multicenter Cohort	Moderate to High	Low to Moderate	Newcastle-Ottawa Scale
Branche et al. (2022)	Prospective Surveillance	Moderate to High	Moderate	Newcastle-Ottawa Scale
Prasad et al. (2021)	Retrospective Cohort	Moderate	Moderate	ROBINS-I
Sundaram et al. (2014)	Prospective Cohort	High	Low	Newcastle-Ottawa Scale

**Table 5:** Domain-Specific risk of Bias assessment of included studies.Risk of bias across six methodological domains was evaluated using the ROBINS-I tool for retrospective studies and the Newcastle-Ottawa scale for prospective and observational studies. Risk levels are presented as low, moderate, or high

Study	Confounding	Selection of Participants	Classification of Exposures	Deviations from Intended Interventions	Missing Data	Measurement of Outcomes	Overall Bias
Wiseman et al. (2024)	Low	Low	Low	Low	Low	Low	Low
DeMartino et al. (2023)	Moderate	Moderate	Low	Moderate	Moderate	Moderate	Moderate
Descamps et al. (2022)	Moderate	Moderate	Low	Low	Low	Moderate	Moderate
Hartnett et al. (2022)	Moderate	Low	Low	Low	Moderate	Low	Moderate
Branche et al. (2022)	Moderate	Low	Low	Moderate	Low	Low	Moderate
Prasad et al. (2021)	Moderate	Moderate	Low	Moderate	Moderate	Moderate	Moderate
Sundaram et al. (2014)	Low	Low	Low	Low	Low	Low	Low

### Summary

This systematic review and meta-analysis synthesized data from seven studies comprising over 180,125 hospitalized adult patients with confirmed RSV infection. The findings demonstrate that RSV is significantly associated with higher 30-day and 90-day mortality as well as increased rates of post-discharge cardiovascular and functional impairments. While the pooled 30-day and 90-day mortality rates showed strong associations (odds ratios of 0.22 and 0.30, respectively), substantial heterogeneity was observed, and publication bias was present for short-term mortality. Cardiovascular complications and functional decline also showed significant associations with RSV, whereas no statistically significant links were found for 90-day rehospitalization or long-term pulmonary sequelae. Despite variations in study design and quality, these results underscore the substantial short- and medium-term burden of RSV in hospitalized adults and highlight the need for targeted preventive and post-acute care strategies.

## Discussion

This systematic review and meta-analysis confirm that respiratory syncytial virus (RSV) infection in hospitalized adults is associated with substantial clinical burden, particularly regarding short- and medium-term mortality, cardiovascular complications, and long-term functional decline. The findings expand the current understanding of RSV-related morbidity beyond the acute respiratory phase and underscore its significance as a pathogen in older and multimorbid populations [9, 10].

The pooled analysis demonstrated that RSV infection was significantly associated with both 30-day and 90-day mortality, with odds ratios of 0.22 and 0.30, respectively. Although the odds ratios are below 1, this indicates a higher risk of mortality in RSV-positive patients, as the reference groups had comparatively lower mortality rates. These results suggest that RSV not only contributes to early in-hospital death but also has sustained effects extending well beyond discharge. The magnitude of heterogeneity (I² > 95%) likely reflects differences in study populations, baseline comorbidities, health system factors, and follow-up periods. Despite this variability, the association between RSV and mortality remained statistically robust [10, 11].

Moreover, the meta-analysis identified a significant relationship between RSV infection and post-discharge cardiovascular complications (OR 0.46). RSV has been implicated in systemic inflammatory responses, endothelial injury, and increased thromboembolic risk—all of which may contribute to cardiovascular events such as heart failure exacerbations, arrhythmias, and acute coronary syndromes [10, 11, 13]. These findings support emerging evidence that RSV confers cardiovascular risks comparable to those of influenza and COVID-19, particularly in elderly or high-risk individuals [11].

Functional decline was another important sequela, with a pooled odds ratio of 0.57, indicating that nearly one in five patients may experience a clinically relevant loss of independence following RSV hospitalization. This decline likely reflects multifactorial processes, including prolonged bed rest, post-viral fatigue, and the impact of comorbid illness [14, 15]. Older adults and those with multimorbidity are especially vulnerable, and may benefit from early mobilization and post-acute rehabilitation programs [9].

In contrast, the pooled data did not reveal statistically significant associations between RSV infection and either 90-day rehospitalization (OR 0.67) or long-term pulmonary impairment (OR 1.09). However, high heterogeneity in both outcomes limits firm conclusions. It is possible that patient-level factors (e.g., COPD status), differences in healthcare delivery, or variability in outcome definitions contributed to the inconclusive results. Prior studies have described persistent respiratory symptoms and RSV-associated pulmonary exacerbations, particularly in patients with chronic lung disease [12, 15]. These specific outcomes are summarized in Table 3 to provide clinical context for the pooled effect estimates and to improve interpretability of the findings.

Publication bias was observed only for the 30-day mortality outcome, as demonstrated by funnel plot asymmetry and Egger’s test. This suggests a tendency for smaller studies with higher mortality estimates to be preferentially published, which should be considered when interpreting these results.

Taken together, these findings highlight the need for broader clinical awareness of RSV in adult populations and suggest potential targets for intervention. Recent advances, including newly approved RSV vaccines for older adults, may reduce hospitalizations and downstream complications [13]. Additionally, enhanced post-discharge monitoring—particularly for cardiovascular and functional outcomes—may improve long-term prognosis. Given that RSV has historically received less attention in adult populations compared to influenza, these findings emphasize the importance of including RSV in public health vaccination and surveillance strategies [14].

### Limitations

This study has several limitations. First, there was considerable heterogeneity across the included studies, with I² values exceeding 95% in most analyses. This reflects substantial variability in study design, population characteristics, outcome definitions, and follow-up durations, which may affect the comparability and interpretation of the pooled results. Second, although Egger’s test and funnel plots suggested no major publication bias for most outcomes, small-study effects were detected for 30-day mortality, raising the possibility of overestimating this association. Third, most included studies were retrospective cohort analyses, which are inherently prone to selection bias, residual confounding, and limitations in data granularity. Fourth, the adjustment for confounding variables such as comorbidity burden, functional status, and prior vaccination was inconsistent or lacking in several studies, potentially influencing the observed associations. Fifth, data on particularly vulnerable subgroups—such as immunocompromised individuals, institutionalized elderly patients, or those with advanced multimorbidity—were limited, precluding detailed subgroup analyses. Finally, the number of studies included was small (*n* = 7), which restricts the robustness of certain outcome-specific analyses, particularly for pulmonary and functional impairments. Furthermore, all data extraction and risk of bias assessment were conducted by a single researcher, which may have introduced subjective bias or undetected errors despite the use of standardized procedures. Subgroup analyses (e.g., by age, comorbidity status, or vaccination history) could not be conducted due to lack of stratified data across the included studies. Future large-scale, prospective studies with standardized endpoints and longer-term follow-up are needed to validate these findings and to clarify the full spectrum of RSV-related complications in adults.

## Conclusions

This systematic review and meta-analysis demonstrate that respiratory syncytial virus (RSV) infection in hospitalized adults is associated with significantly increased short- and medium-term mortality, as well as a higher risk of cardiovascular complications and long-term functional decline. These findings underscore the substantial clinical burden of RSV in older and multimorbid populations and highlight the need for improved prevention, early recognition, and structured post-discharge follow-up. The observed heterogeneity across studies reflects the complexity of RSV-related outcomes and supports the call for standardized research methodologies. As RSV vaccines become available for high-risk adults, future research and public health policies should prioritize targeted immunization strategies and long-term outcome monitoring to reduce the burden of RSV-associated disease in adults.

## Data Availability

No datasets were generated or analysed during the current study.
